# In search for geroprotectors: in silico screening and in vitro validation of signalome-level mimetics of young healthy state

**DOI:** 10.18632/aging.101047

**Published:** 2016-09-24

**Authors:** Alexander Aliper, Aleksey V. Belikov, Andrew Garazha, Leslie Jellen, Artem Artemov, Maria Suntsova, Alena Ivanova, Larisa Venkova, Nicolas Borisov, Anton Buzdin, Polina Mamoshina, Evgeny Putin, Andrew G. Swick, Alexey Moskalev, Alex Zhavoronkov

**Affiliations:** ^1^ Insilico Medicine, Inc, Research Department, Baltimore, MD 21218, USA; ^2^ Moscow Institute of Physics and Technology, Dolgoprudny, 141700, Russia; ^3^ Center for Biogerontology and Regenerative Medicine, Moscow, 121099, Russia; ^4^ Genetics, Genomics, and Informatics, University of Tennessee Health Science Center, Memphis, TN 38163, USA; ^5^ D. Rogachev Federal Research and Clinical Center for Pediatric Hematology, Oncology, and Immunology, Moscow, 117997, Russia; ^6^ Laboratory of Molecular Radiobiology and Gerontology, Institute of Biology of Komi Science Center of Ural Branch of Russian Academy of Sciences, Syktyvkar, 167982, Russia; ^7^ Pathway Pharmaceuticals, Ltd, Hong Kong, Hong Kong; ^8^ Life Extension, Ft. Lauderdale, FL 33308, USA; ^9^ School of Systems Biology, George Mason University (GMU), Fairfax, VA 22030, USA; ^10^ Engelhardt Institute of Molecular Biology of Russian Academy of Sciences, Moscow, 119991, Russia; ^11^ The Biogerontology Research Foundation, Oxford, UK

**Keywords:** geroprotector, drug discovery, screening, drug repurposing, aging

## Abstract

Populations in developed nations throughout the world are rapidly aging, and the search for geroprotectors, or anti-aging interventions, has never been more important. Yet while hundreds of geroprotectors have extended lifespan in animal models, none have yet been approved for widespread use in humans. GeroScope is a computational tool that can aid prediction of novel geroprotectors from existing human gene expression data. GeroScope maps expression differences between samples from young and old subjects to aging-related signaling pathways, then profiles pathway activation strength (PAS) for each condition. Known substances are then screened and ranked for those most likely to target differential pathways and mimic the young signalome. Here we used GeroScope and shortlisted ten substances, all of which have lifespan-extending effects in animal models, and tested 6 of them for geroprotective effects in senescent human fibroblast cultures. PD-98059, a highly selective MEK1 inhibitor, showed both life-prolonging and rejuvenating effects. Natural compounds like N-acetyl-L-cysteine, Myricetin and Epigallocatechin gallate also improved several senescence-associated properties and were further investigated with pathway analysis. This work not only highlights several potential geroprotectors for further study, but also serves as a proof-of-concept for GeroScope, Oncofinder and other PAS-based methods in streamlining drug prediction, repurposing and personalized medicine.

## INTRODUCTION

A significant rise in the proportion of seniors worldwide is underway [1, 2], resulting in increasing rates of chronic, debilitating disease and long term residential care [3], shrinking the supporting workforce [1, 2], and threatening to sink current health care systems. Prevention will be crucial moving forward. If aging can be delayed and diseases prevented, productive years can be extended and retirement age redefined. A shift in focus from “last mile” treatments to longevity via prevention may not only skirt economic collapse but also spell unprecedented economic growth [4]; thus the search for anti-aging interventions has never been so important.

Anti-aging therapies have been sought since the dawn of human civilization, but with the rise of modern biology, big data, and information sciences, intelligent approaches to geroprotector discovery may finally be within reach. Aging is a complex multifactorial process involving many often-intersecting pathways, with effects uniquely manifested in each tissue and cell type throughout an organism [5–9]. Aging research is thus highly multidisciplinary [9, 10]. While many questions remain unanswered, many details have been elucidated and aging theories proposed [11]. The outward features of aging, including decline in function and rise in susceptibility to stress and disease, are associated with a set of structural and functional changes at the cellular level. While these changes vary by tissue, many are genetically regulated, and many genes mediating longevity, termed gerontogenes, have been identified [5]. The identification of these genes and experimental manipulation of their products to extend lifespan in model organisms [12] has bolstered the notion that aging is not just a natural process but a treatable disease [8, 13, 14] and added credence to the movement to identify drugs or other factors that may also extend lifespan, or, more favorably, healthspan, in humans. These are termed geroprotectors.

There are now over 200 substances that have shown geroprotective effects in model organisms; these are continuously indexed at geroprotectors.org [15]. Human-based studies, however, may turn out to be more productive. Several of the most promising attempts at developing geroprotectors have involved identifying FDA-approved drugs with life-extending qualities and repurposing them as geroprotectors for human use. These include rapamycin [16] and metformin [17–19]. However, a number of problems still hamper the widespread approval and use of these or other drugs for this purpose [20]. Most notably, longevity is a difficult parameter to study in humans without large, longitudinal designs, and since these drugs would presumably be administered to aging but otherwise healthy individuals, the effect size would have to be substantial and side effects almost non-existent. In addition, the FDA does not consider aging an approved disease indication. At this time, no drug has sufficiently met these conditions, and new approaches to drug discovery - and drug repurposing - are needed.

The drug discovery process is slow and expensive, burdened by many projects that dead-end before clinical trial or fail thereafter [21, 22]. Improved prediction of drug performance prior to lengthy experimentation would cut waste [21, 22]. Vast datasets now exist that enable such prediction with the help of sophisticated computational methods [23, 24]. Two particularly valuable datasets in this respect are the literally millions of gene expression profiles stored in repositories such as GEO [25, 26] and a number of increasingly diverse compound screening libraries [27]. While gene expression data can be used to pinpoint target pathways for a particular disease, compound libraries can be screened for drugs that target these pathways. All of this can be done in silico, at relatively little cost.

Recently, a method was developed that would do just this - capitalize on existing gene expression data and compound libraries to improve prediction of targeted drugs [28, 29]. The method involves the use of an algorithm termed Oncofinder [29], which performs advanced signaling pathway analysis of gene expression data. Signaling pathways play a vital role in every biological process, including the process of aging. Characterizing pathway activation can elucidate mechanisms of aging and anti-aging interventions; for example, the lifespan-extending effects of pectins in fruit flies have been closely tied to increases in NF-κB signalling and activation of stress resistance genes [30].

Oncofinder quantifies Pathway Activation Strength (PAS) in a given sample based on gene expression patterns relative to another sample. Thus PAS values can be computed for a disease state in comparison to a normal state, old versus young, or any other set of physiological conditions. The net changes in pathways in a given condition, or pathway cloud, can then be used as a profile against which compound libraries can be screened for substances that would best restore it to normal levels, based on their known targets [28,29]. A shortlist of candidate substances can then be compiled and experimentally validated *in vitro* to select best candidates for further study.

Here, we used an aging-based extension of Oncofinder, known as GeroScope [28], in a search for novel geroprotective substances. Using GEO datasets, we first quantified activation of age-related pathways in hematopoietic and mesenchymal stem cells from “old” (vs “young”) human donors. We then shortlisted substances predicted to best target those pathways, restore a “young” cellular profile, and extend viability. From that list, we proceeded to experimentally test the effects of each substance in human fibroblasts.

## RESULTS

### Profiling of database-extracted transcriptional data with GeroScope algorithm

To develop a signature of age-related signaling pathway activation and rank candidate geroprotectors, we applied the GeroScope algorithm[28] to preprocessed transcriptional data extracted from 57 bone-marrow derived human hematopoietic and mesenchymal stem cell samples (see Methods for details). Pathway activation scores were calculated for “old” samples (donor over 60 years of age) compared to “young” (donor between 15 and 30 years of age). Then drug GeroScore ratings were calculated from a database of known geroprotectors and their targets (Supplementary Table S2).

The top ten candidate anti-aging compounds, based on GeroScores, were selected for further testing; these are listed in Table 1.

**Table 1:** Letter codes for the test conditions

Cells	Substance	Code
young	-	Y
old	-	O
old	Nordihydroguaiaretic acid (NDGA)	A
old	Myricetin	B
old	HA-1004	*C*
old	7-Cyclopentyl-5-(4-phenoxy)phenyl-7H-pyrrolo[2,3-d]pyrimidin-4-ylamine	*D*
old	Staurosporine	*E*
old	Ursolic acid	*F*
old	N-acetyl-L-cysteine (NAC)	G
old	Fasudil (HA-1077)	H
old	PD-98059	I
old	Epigallocatechin gallate (EGCG)	J

### Incubation with test substances

To verify the predictive potential of the GeroScope algorithm, the substances suggested by the program were added to non-transformed human embryonic lung fibroblasts at the senescence stage (“old”) in 50 μM concentrations and incubated for 3 days. Fibroblasts from several passages earlier, in a pre-senescent state (“young”) served as control. The test conditions (cells+substance) were coded with letters A-J (Table 1).

Of the ten substances listed, four were excluded from further analysis. HA-1004 was excluded because it was unavailable. Cells in the 7-Cyclopentyl-5-(4-phenoxy)-phenyl-7H-pyrrolo[2,3-d]pyrimidin-4-ylamine, Staurosporine and Ursolic acid flasks died prior to the main experiment; therefore, these conditions were also excluded.

### Flow cytometry

**Figure 1:**
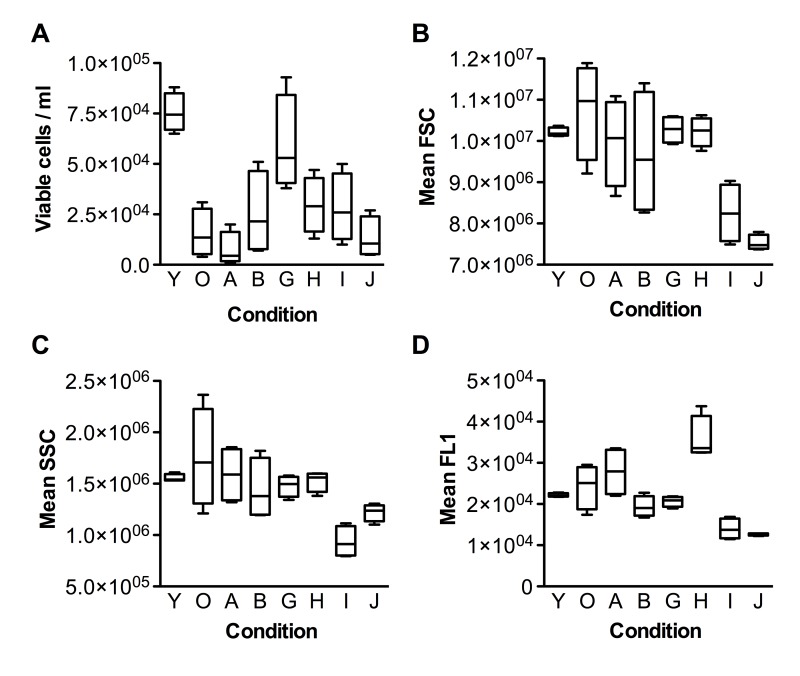
Flow cytometric characterization of fibroblasts upon incubation with the test substances (**A**) Cell viability, (**B**) FSC (Forward-scattered light) - cell size metric, (**C**) SSC (Side-scattered light) - granularity metric, (**D**) FL1 - fluorescence metric. Group codes are listed in Table 1.

It is known that older (senescent) cells are typically larger than younger ones; they also contain more lysosomes and mitochondria, exhibit increasing cell granularity, and accumulate lipofuscin, which leads to increase in cell autofluorescence [31]. Thus, flow cytometry is an ideal tool to investigate the senescence of a cell population. After 3 days of incubation with the test substances, cells were lifted from flasks and analyzed with a flow cytometer. Viable cells were gated according to forward scatter (FSC) and side scatter (SSC) parameters, and then their concentration, size (FSC), granularity (SSC) and autofluorescence (FL1) were recorded (Figure 1).

As can be seen from Fig. 1A, the fibroblast culture at senescent stage (condition O) had much fewer viable cells than the pre-senescent one (condition Y). Most of the test substances slightly increased the viability of senescent cells, with the exception of Nordihydroguaiaretic acid (NDGA), which decreased it. Interestingly, N-acetyl-L-cysteine (NAC) increased viability to nearly the level of pre-senescent cells. As expected, cells in the senescent culture were typically bigger than pre-senescent ones, and with larger variation in size (compare Y and O in Fig.1B). All test substances decreased the mean size of senescent cells, and most of them also decreased the variation in cell size. Notably, PD-98059 and Epigallocatechin gallate (EGCG) decreased the mean size of senescent cells (SSC) much below the size of the pre-senescent control. The changes in cell granularity were comparable to the changes in cell size (Fig.1C), with the exception of PD-98059, which had a stronger effect than EGCG. Autofluorescence of senescent cells also behaved similarly to cell size, except that Fasudil unexpectedly increased autofluorescence (Fig.1D).

### Beta-galactosidase staining

**Figure 2:**
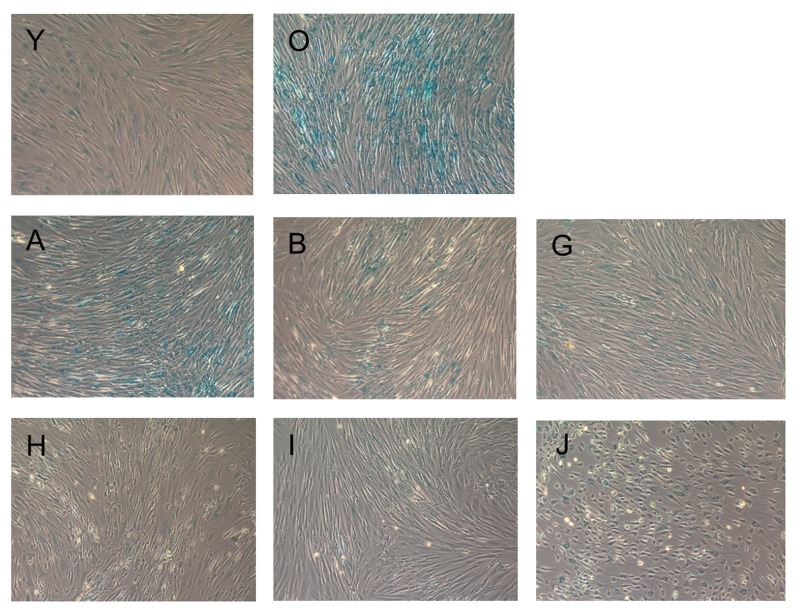
Beta-galactosidase staining of fibroblasts upon incubation with the test substances Blue staining indicates cellular senescence. Images are named according to the letter code of the substance provided in Table 1.

To measure the effect of the substances on cellular senescence, we followed the conventional method for determining cellular senescence, staining for senescence beta-galactosidase activity at pH6 [32]. The results of staining are presented in Figure 2.

Compared to condition O (senescent cells), all substances, except NDGA, strongly reduced beta-galactosidase staining of senescent fibroblasts. PD-98059 had the most pronounced effect. Interestingly, Fasudil and EGCG changed the cell morphology to neuron-like (see Supplementary Figure S1 for higher magnification).

### Long-term survival

**Figure 3:**
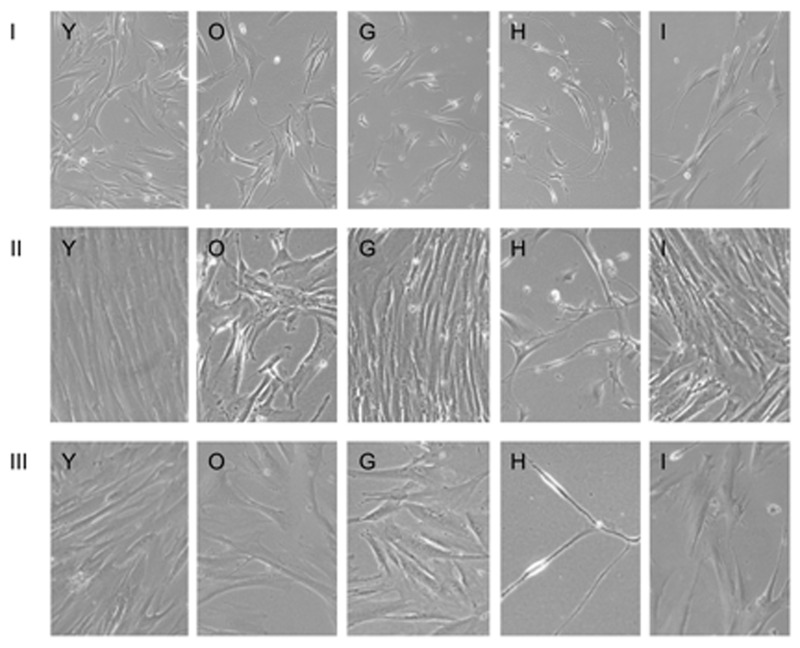
Long-term culture of fibroblasts in the presence of test substances for I, II and III passage Images are named according to the letter code of the condition/substance provided in Table 1.

To determine the effects of the test substances on the long term survival and division capabilities of senescent fibroblasts, we incubated the cells for 3 more passages (18 days). With every change of the medium, test substances were added again. The morphology and density of the cells after the 1st, 2nd and 3rd passage can be seen in Figure 3. Cells in the presence of NDGA, Myricetin and EGCG substances died prior to the 1st passage, so are not shown.

By the 2nd passage, senescent cells in the presence of substances G and I divided as well as pre-senescent cells (Y). Meanwhile, senescent cells in the presence of Fasudil and those without any additives (O) divided poorly. Cells in the presence of Fasudil retained neuron-like morphology. By the 3rd passage all cells slowed proliferation, and cells in the presence of Fasudil became thread-like.

The effects of the test substances on the senescent fibroblasts are summarized in Table 2. As can be seen from the table, NDGA had almost no effect on senescent phenotype, but decreased both short- and long-term survival. Myricetin had mild rejuvenating effect as judged by cell phenotype, but severely compromised long-term survival. NAC had a very mild rejuvenating effect but dramatically increased short- and long-term survival. Fasudil also had very mild rejuvenating effect but did not dramatically affect survival. However, it induced strong autofluorescence and neuron-like morphology. PD-98059 had a very strong rejuvenating effect and increased both short- and long-term survival. Finally, EGCG also had very strong rejuvenating effect but induced neuron-like morphology and dramatically decreased long-term survival. Overall, these results indicate that PD-98059 possesses the strongest rejuvenating and pro-survival properties of the substances tested.

**Table 2:** The effects of the test substances on the senescent fibroblasts

Code	Name	Viability	Size	Granularity	Auto-fluorescence	Beta-galactosidase	Morphology	Survival
A	NDGA	-	-/=	-/=	+/=	-/=	normal	---
B	Myricetin	+/=	-	-/=	-	--	normal	---
G	NAC	+++	-/=	-/=	-/=	--	normal	+++
H	Fasudil	+	-/=	-/=	+++	--	elongated neuron-like	-
I	PD-98059	+	--	---	--	---	normal	+++
J	EGCG	=	---	--	---	--	round neuron-like	---

To investigate the mechanism of action of these compounds we performed pathway analysis. For this purpose we utilized transcriptional response data provided from Library of Integrated Network-based Cellular Signatures (LINCS) L1000 dataset. After data processing (see Methods) we obtained pathway activation scores for 97 age-related pathways (Supplementary Table S4). EGCG showed strong upregulation of cAMP pathway and inhibition of mitochondrial apoptosis and Ras pathways. Myricetin was found to upregulate ILK, DNA repair, cAMP and Hypoxia pathways. On the other hand, it severely suppressed PAK, IL-6, MAPK, Cellular senescence, p38, mTOR and several chemokine pathways. NAC showed strongly inhibition of pro-proliferative pathways like MAPK, AKT, p38, RAS, PAK, ERK and in turn activated p53, EGFR1, SMAD and Caspase signaling.

Additionally, to evaluate potential side effects of top scored geroprotectors we used a set of deep neural networks, trained on drug-induced transcriptional response data (see Methods). We estimated the probability of 205 side effect classes for 8 compounds from this study (Supplementary Table S5). Ursolic acid was predicted to have the most side effects collecting 38 different classes with probability more than 0.9. These include gastrointestinal, vascular and muscle disorders. EGCG was found to have the smallest number of probable side effects, comprising only 7 common categories that are shared amongst all 8 geroprotectors.

## DISCUSSION

A major goal of aging research has been to identify and develop geroprotectors that increase healthspan by delaying the onset of aging and disease, but geroprotector development has been slow and mostly limited to lifespan extension in animal models. Improved in silico prediction of geroprotectors (and active compounds in other fields) is necessary and now possible, and the combination of prediction with *in vitro* validation in human cell lines may be a more promising path forward.

Here, we used GeroScope, a signaling pathway activation-based algorithm, to screen and rank known substances based on their predicted ability to mimic the signalome of young (vs old) human donors. We then tested the top-scoring substances’ effects in human fibroblasts. The versatile PAS scoring approach of GeroScope and Oncofinder has already shown useful in a variety of applications, including defining biomarkers for cancer [33] and signatures of signaling pathway activation for asthma [34] and primary and metastatic melanoma [35]. It has been used to analyze parallels in pathway activation between pathological and chronological aging in Hutchinson-Gilford Progeria Syndrome fibroblasts, a disease-based model of aging [36]. It has also been used in evaluating the therapeutic viability of pluripotent stem cells [37] and in cross-species analysis [38], an important aspect of aging research [30]. Additionally it has helped determine common pathway signatures in lung and liver fibrosis [39] and evaluate pro-fibrotic pathway activation in trabecular meshwork and lamina cribrosa in glaucoma [40]. It has even been applied in the financial sector [41] and for model reduction for deep learning applications [42, 43]. Perhaps its greatest potential, however, may be in the area of drug discovery and personalization, where its ability to aid prediction is projected to streamline these processes significantly [28, 29, 44-46].

The ten test substances selected from GeroScope output have all previously been shown to extend lifespan in animal models (Table 3) [15]. They vary from FDA-approved drugs to naturally occurring dietary substances. FDA-approved drugs included nordihydroguaiaretic acid (NDGA, aka masoprocol), a drug tested in humans for treatment of prostate cancer [56] and in mice in the National Institute on Aging Interventions Testing Program (ITP) [57], where it increased lifespan in males [48], and N-acetyl-L-cysteine, an FDA-approved drug and dietary supplement with a large and highly variable list of current and potential human applications, from treating acetaminophen overdose [58] to various neuropsychiatric disorders [59]. Another drug on the list, Fasudil (HA-1077), is not yet approved in the US but is used abroad in stroke treatment [60] and is currently under investigation for cognitive enhancement in aging [61] and possible anti-inflammatory-based protection against Aβ-induced hippocampal neurodegeneration in Alzheimer's disease [62]. Non-drug, plant-derived compounds included Myrecitin, a naturally occurring flavonol present in fruits, vegetables, nuts, and berries [49], and Epigallocatechin gallate, a catechin found in green tea [54, 55].

**Table 3:** Previously reported lifespan effects of test substances in animal models (compiled from geroprotectors.org [15].)

Drug	Code	Model Organism	Lifespan (LS) Parameter	% Increase	Ref.
Nordihydroguaiaretic acid	A	D. melanogaster	Median LS	23	[47]
		Mus Musculus	Median LS	12	[48]
Myricetin	B	C. elegans	Mean LS	32.9	[48. 49]
HA-1004	*C*	D. melanogaster	Mean LS	18	[50]
7-Cyclopentyl-5-(4-phenoxy)phenyl-7H-pyrrolo[2,3-d]pyrimidin-4-ylamine	*D*	C. elegans	Mean LS	11	[51]
Staurosporine	*E*	D. Melanogaster	Mean LS	34.8	[50]
Ursolic acid	*F*	C. elegans	Mean LS	39	[52]
N-acetyl-L-cysteine	G	Mice	Max LS	40	[53]
Fasudil (HA-1077)	H	D. melanogaster	Mean LS	14.5	[50]
PD-98059	I	D. melanogaster	Mean LS	27	[50]
Epigallocatechin gallate	J	C. elegans	Mean LS	10.1	[54]
		Rattus norvegicus	Median LS	13.5	[55]

We evaluated the performance of each of these substances in human fibroblasts, looking for enhanced viability as evidence of life-prolonging qualities and reduction in cellular size, granularity, and senescence-based staining as evidence of mimicry of or rejuvenation to a younger cellular state.

Most of the geroprotectors tested complied with the recently published criteria for geroprotector [20] The top geroprotector, in terms of performance in both enhancing viability and rejuvenation was PD-98059, a highly selective inhibitor of MEK1 and the MAP kinase cascade [63]. MEK inhibition along with PI-3K inhibition has been shown to decelerate cellular senescence via the mTOR/S6 pathway, a known target for anti-aging interventions [64], although not with PD-98059 [65]. PD-98059 is anti-proliferative in colorectal cancer when combined with rapamycin [65, 66]. It has also shown therapeutic potential in atherosclerotic disease [67] and Alzheimer's disease, preventing fibrillar Aβ-induced tau phosphorylation and neurite degeneration in mature hippocampal neurons and highlighting the importance of MAPK signal transduction in that process [68]. MAPK is one of the most important pathways in replicative senescence. It mediates the induction of p16^INK4A^, the key biomarker and regulator of cellular senescence [69], and was recently targeted to successfully reverse the aging phenotype of klotho mice [70].

Aside from PD-98059, most of the studied geroprotectors had effects on either cellular viability or senescence features. The most significant effects with potential synergy were observed for NAC, Myrecitin and Epigallocatehin gallate.

### Synergistic effects

EGCG is a known flavonoid antioxidant with anti-cancer [71] and anti-diabetic [72] properties. On the pathway level it showed strong activation of cAMP pathway, which was recently found to induce anti-aging effects characteristic of caloric restriction via up-regulation of sirtuins [73]. Here we show that EGCG decreased the cell size, granularity and fluorescence of replicatively senescent fibroblasts.

NAC is known to protect cells from stress and inhibit inflammation through suppression of NF-kB, COX-2 and several pro-inflammatory cytokine pathways [74, 75]. The NAC geroprotective action predicted and observed in this paper is in accordance with several other studies showing its positive effect on the senescence of other cell models of induced senescence [76–78]. Pathway analysis performed here confirmed its anti-senescent properties as it inhibited pro-proliferative MAPK, p38, AKT, PAK, ERK signalling and activated p53 pathway. Among tested compounds, NAC showed the best performance in terms of cell viability, reaching the properties of young fibroblasts.

The natural flavonoid Myricetin is considered to be a very potent antioxidant, anti-inflammatory and anti-neoplastic agent [79]. Directly interacting with tyrosine kinase receptors, particularly JAK1, it influences Insulin receptor, EGFR and AR signaling [80, 81]. To the best of our knowledge, this is the first demonstration of geroprotective properties of Myricetin in human replicatively senescent cells. Here we showed that on the pathway level it strongly inhibits PAK, MAPK, mTOR, cellular senescence and several chemokine pathways. PAK pathway activation was linked to premature senescence via aforementioned p16INK4A and MAPK cascade [82], hence its down-regulation may be very beneficial. Myricetin also activates ILK, Hypoxia and, similar to EGCG, cAMP signaling.

Each of these three compounds investigated on the pathway level covers a particular side of the senescence process and some of the effects are shared among compounds: EGCG and Myricetin both activate cAMP pathway; Myricetin and NAC inhibit pro-proliferative signaling via MAPK, p38, PAK and AKT signaling, whereas the effect of NAC on these pathways was stronger. The combination of these compounds with proper dosing may reveal synergistic effects and turn out to be even more beneficial than independent use.

On top of pathway analysis, the predicted safety of investigated compounds was evaluated with deep learned side effects prediction approach. It predicted that the most harmless compound out of investigated geroprotectors was EGCG, while Ursolic acid comprised the highest number of probable side effects.

This study thus not only demonstrated geroprotective effects of several known substances but also highlighted a new approach to geroprotector prediction and discovery with a screening, validation and safety estimation of new geroprotectors, illustrating the potential value in pathway analysis, PAS-based techniques and deep learning in the areas of drug discovery, drug repurposing, and personalized medicine.

## MATERIALS AND METHODS

### GeroScope algorithm and software

The transcriptomic data for bone-marrow derived human hematopoietic and mesenchymal stem cells were extracted from GEO datasets GSE32719 and GSE39540, respectively. These datasets were profiled on Affymetrix Human Genome U133 Plus 2.0 Array and Affymetrix Human Genome U133A 2.0 Array, respectively All samples gathered from these datasets were divided into two groups: “young” and “old,” according to donor age, with “young” donors ranging from 15-30 years of age and “old” donors over 60 years of age. Young and old groups consisted of 14 and 8 samples from GSE32719 and 7 and 28 samples from GSE39540, respectively. Each preprocessed gene expression dataset was independently analyzed using an algorithm called OncoFinder [29] implemented in a new platform for analyzing signaling pathways in aging called GeroScope [28]. The signaling pathways associated with aging were constructed manually from available literature[5] and partly came from the database OncoFinder utilized in previous studies [35, 36]. Results for the 97 age-related pathways were obtained for each sample (listed in Supplementary Table S1).

The database of geroprotector drugs with their molecular targets used in this study consists of 70 compounds (Supplementary Table S2). Predicted geroprotective efficacy of the drug (GeroScore, GS) is calculated as follows:

$$GS_{d}=\sum_{t}DTI_{dt}\sum_{p}NII_{tp}gARR_{t}gPAS_{p}gPAR_{p}$$

where d – drug, t – protein target, p – signaling pathway.

Drug-target index (DTI) equals to −1, 1 and 0 if drug activates, inhibits or does not interact with protein target t, respectively. Node involvement index (NII) is a boolean variable indicating if target t is present in the pathway p (TRUE) or not (FALSE). Activator/repressor role (ARR) is indicative of the role of protein t in the pathway as described in [29]. Pathway aging role (PAR) equals to 1 and −1 for pro- and anti-aging pathways, respectively. GeroScore ratings were then calculated for “old” individual transcriptomes as compared to young (Supplementary Table S3).

For pathway analysis of several selected compounds we utilized transcriptional response data provided by LINCS Project (http://www.lincsproject.org/). We extracted the level 3 (Q2NORM) gene expression data for PC3 cell line perturbed with 10 uM concentration of each compound independently for 6 hours. In the pathway level analysis, for each given case sample group perturbed with a compound, we generated a reference group consisting of samples perturbed with DMSO that came from the same RNA plate as samples from the case group. After that, each case sample group was independently analyzed using an algorithm called OncoFinder. Taking the preprocessed gene expression data as an input, it allows for cross-platform dataset comparison with low error rate and has the ability to obtain functional features of intracellular regulation using mathematical estimations. For each investigated sample group it performs a case-reference comparison using Student's t-test, generates the list of significantly differentially expressed genes and calculates the Pathway Activation Strength (PAS), a value which serves as a qualitative measure of pathway activation. Positive and negative PAS values indicate pathway up- and down-regulation, respectively. In this study the genes with FDR-adjusted p-value<0.05 were considered significantly differentially expressed. Samples with zero pathway activation score for all of the pathways were considered as insignificantly perturbed and were excluded from further analysis.

Deep neural networks (DNNs) were trained with transcriptional response data from LINCS L1000 dataset. Side effects for drugs were derived from SIDER database [83]. Side effect categories were mapped onto 205 preferred terms from MedDRA v16.0 ontology [84]. An ensemble of class-specific DNNs with binary output was trained in a similar way to the methodology described previously [85]. Similarly to pathway analysis section, for prediction we chose samples of gene expression data for PC3 cell line perturbed with 10 uM concentration (or 70.07 uM in case of ursolic acid) of compound independently for 6 hours. Resulting side effect probabilities were averaged across replicates.

### Cell culture

Non-transformed human embryonic lung fibroblast cell line FLECH-104 was purchased at 20th passage in Biolot (Saint-Petersburg, Russia, #1.5.9.1). Cells were cultured in 75 cm2 Nunc EasYFlasks (Thermo Scientific, #156472) in EMEM medium with L-glutamine and double amino acids (Biolot, #1.3.13), 10% of HyClone fetal bovine serum (GE Healthcare, #SV30160.03) and 50 μg/ml of gentamycin (Biolot, #1.3.16). Flasks with 20 ml of growth medium were incubated at 37°C and 5% CO2. Every 6 days, when cells nearly achieved monolayer, they were lifted with Trypsine-Versene (EDTA) (Biolot, #1.2.7) and passaged onto new flasks in a 1:4 ratio. Cells were cultured as described until the irreversible division block (27th-28th passage).

### Cell freezing and thawing

During cell culture, part of the cells from each passage was frozen in liquid nitrogen, as follows. Cells lifted with Trypsine-Versene were centrifuged at 100xg for 5 min and resuspended at 106 cells/ml in cold growth medium with 20% of serum and 10% of DMSO (Biolot, #1.4.7). Then the suspension was aliquoted to 1 ml cryotubes, which were placed in a room-temperature Mr. Frosty container (Thermo Scientific, #5100-0001). The container was then incubated at −80°C for 4 hours, and cryotubes were placed in Locator 6 Plus cryostorage system (Thermo Scientific, #CY509109) directly above liquid nitrogen (in gas phase). When required, aliquots were thawed by placing in a water bath at 37°C and intensive shaking for 2 minutes. They were then immediately mixed with 19 ml of prewarmed growth medium and placed in an incubator. On the next day, upon cell attachment, the medium was replaced with a fresh one.

### Experiment preparation

To generate senescent cells (“old”), 18 days before the date of the main experiment, frozen aliquots from the end of 23rd passage were thawed, and cells were plated on flasks. After 6 days, cells were lifted, combined and plated on flasks 1:4. After another 6 days, the cells were again lifted, combined and plated 1:4 on flasks and 6-well plates. As a result, on the day of the main experiment cells were at the end of the 26th passage (approximately 52 population doublings).

To generate pre-senescent cells (“young”), 9 days before the date of the main experiment frozen aliquots from the end of the 22nd passage were thawed, and cells were plated on flasks. After 6 days, cells were lifted, combined and plated 1:2 on flasks and 6-well plates. As a result, on the day of the main experiment cells were at the middle of the 24th passage (approximately 47 population doublings).

3 days before the date of the main experiment, 20 mM stock solutions of the test substances (all from Sigma Aldrich) in DMSO (or DMSO alone as control) were added to the cells in a 1:400 dilution. Thus, the final concentration of the test substances was 50 μM and final concentration of DMSO was 0.25%. Each substance was tested in 4 replicates.

### The senescence monitoring experiment

On the day of the experiment, cells in each flask were lifted and resuspended in 20 ml of growth medium. 1 ml of suspension was analyzed in Accuri C6 flow cytometer (BD Biosciences, #653118). First, viable cells were gated according to forward scatter (FSC) and side scatter (SSC) parameters, and then concentration, size (FSC), granularity (SSC) and autofluorescence (FL1) were recorded. 1 ml of suspension from each replicate flask were combined, mixed with 16 ml of growth medium with 50 μM of the test substance, and plated on the new flask. They were further passaged in presence of the test substance after 6, 12 and 18 days.

Before each passage, flasks were photographed on the Axio Observer A1 microscope with A-Plan 10x/0.25 Ph1 objective, AxioCam MRc5 camera with 0.63x adapter and Zen Pro software (all from Zeiss). Cells on 6-well plates were processed with the senescence beta-galactosidase staining kit (Cell Signaling, #9860) and visualized the next day with the Axio Observer A1 microscope.

## SUPPLEMENRATY MATERIAL TABLES AND FIGURE


