# Supplementary material for: In search for geroprotectors: in silico screening and in vitro validation of signalome-level mimetics of young healthy state
Source: Aging (Albany NY). 2016 Sep 24;8(9):2127–41. doi: 10.18632/aging.101047 (PMC5076455; doi:10.18632/aging.101047)
Supplement: Supplementary file 1 [file aging-08-2127-s001.pdf]

SUPPLEMENRATY MATERIAL

Supplementary Table S1. Pathway activation analysis results for datasets GSE32729 and GSE39540.

	GSE39540		GSE32729	
Pathway name	average	stdev	average	stdev
AKT_Pathway	9.59	4.74	12.75	16.41
Androgen_receptor_Pathway	5.25	3.24	5.55	3.97
Antioxidants	0.88	1.13	2.23	3.38
ATM_Pathway	0.43	0.85	1.24	0.91
Autophagy	-0.42	1.14	0.35	0.71
Base Excision Repair	-0.14	0.51	0.22	0.81
cAMP_Pathway	8.13	4.15	11.29	9.07
Caspase_Cascade	-4.26	2.68	-4.14	4.29
CD40_Pathway	0.26	1.28	0.99	1.66
Cellular Senescence	-0.61	1.21	-1.57	1.82
Cellular_Anti-Apoptosis_Pathway	4.96	3.15	3.50	3.55
Chemokine_Pathway	3.36	2.17	9.55	9.15
Chromatin_Pathway	0.15	0.53	0.65	0.54
Circadian Rhythms	0.38	0.86	0.73	1.00
Circadian_Pathway	-0.08	0.47	0.05	0.99
CREB_Pathway	5.85	3.42	6.26	6.15
Cytokine_Network_Pathway	0.48	0.57	0.86	1.37
DNA damage response	0.14	0.61	0.41	0.69
DNA Methyltransferases	0.02	0.15	0.12	0.25
DNA_Repair_Mechanisms_Pathway	0.99	1.59	1.53	1.74
Double-Strand Break Repair	0.23	0.79	0.77	1.23
EGFR1_Pathway	1.06	2.25	5.10	2.84
eIF4e-p70 S6	-0.02	0.50	0.26	0.56
ER stress response	-0.22	0.50	0.09	0.36
ErbB_Family_Pathway	1.43	1.49	3.46	2.14
ERK_Signaling_Pathway	13.29	6.77	15.71	15.06
Erythropoeitin_Pathway	1.91	2.01	5.59	2.18
Estrogen_Pathway	5.90	3.98	5.54	3.39
Fas_m_Signaling_Pathway	0.29	0.64	0.83	1.04
Fas_p_Signaling_Pathway	0.14	0.66	0.84	1.13
FLT3_Signaling_Pathway	0.45	1.03	2.00	1.93
Glucocorticoid_Receptor_Pathway	2.96	2.87	7.95	9.41
GPCR_Pathway	6.77	2.93	9.30	6.30
Growth_Hormone_Pathway	0.36	1.10	2.44	1.52
GSK3_Pathway	6.85	2.60	6.78	3.94
Heat shock response	0.47	1.00	0.98	0.68
Hedgehog	0.06	0.98	-0.09	0.85

	GSE39540		GSE32729	
Pathway name	average	stdev	average	stdev
Hedgehog_Pathway	0.10	0.61	0.31	0.51
HGF_Pathway	4.06	2.80	4.71	4.83
HIF1Alpha_Pathway	0.68	0.93	1.17	1.42
Hippo	0.09	0.29	0.75	0.88
Histone Deacetylases	0.06	0.78	0.50	0.81
Hypoxia	0.13	0.55	0.48	1.90
Hypoxia-induced_EMT_in_cancer_and_fibrosis_3	0.18	0.47	0.15	0.43
IGF-1	0.55	1.39	1.19	1.12
IGF1R_Signaling_Pathway	0.77	1.30	2.90	2.73
IL_10_Pathway	0.87	1.07	1.49	2.17
IL_2_Pathway	2.03	2.47	7.29	5.09
IL_6_Pathway	2.75	3.32	3.80	6.38
ILK_Pathway	13.72	8.32	15.05	7.54
Inflammation	0.72	1.27	3.59	3.63
Integrin_SIgnaling_Pathway	6.77	4.03	7.73	5.90
Interactions Report	0.01	0.32	0.08	0.76
IP3_Pathway	2.11	2.11	4.19	6.88
JAK_mStat_Pathway	-0.01	0.27	-0.08	0.96
JNK_Pathway	6.40	3.34	10.72	12.27
MAPK_Family_Pathway	3.98	2.62	9.29	11.69
MAPK_Signaling_Pathway	13.59	6.88	15.35	10.76
Mismatch Repair	-0.25	0.71	0.29	0.92
Mismatch_Repair_Pathway	-0.26	1.06	0.10	0.25
Mitochondrial_Apopotosis_m_Pathway	-3.80	2.50	-3.57	3.40
mTOR	-0.07	0.46	0.05	0.23
mTOR_Pathway	4.92	2.62	4.82	3.16
NFkB	0.28	0.94	1.32	1.80
NGF_m_Pathway	-0.15	0.74	0.38	0.55
NGF_p_Pathway	0.05	1.88	2.66	2.69
Notch	0.47	1.05	0.67	0.98
Notch_Pathway	0.33	1.00	0.79	1.51
NRF2 Oxidative Stress Response	0.02	0.15	0.00	0.00
Nucleotide Excision Repair	0.01	0.07	0.00	0.22
Osmotic Stress	0.57	0.77	0.50	0.79
Oxidative Stress Response	0.39	1.03	0.40	0.74
p38_m_Signaling_Pathway	9.78	5.02	17.25	20.03
p53_Signaling_m_Pathway	0.17	1.53	1.11	1.12
PAK_Pathway	6.46	4.14	8.32	9.30
PI3K-AKT	0.20	0.98	-0.18	0.64
Polycomb-Trithorax	0.03	0.87	1.27	0.87
PPAR_Pathway	5.06	2.89	2.82	4.21

	GSE39540		GSE32729	
Pathway name	average	stdev	average	stdev
PTEN_Pathway	-0.32	0.66	-1.66	1.44
RANK_Signaling_in_Osteoclast_Pathway	0.67	1.60	3.22	2.44
RAS_Pathway	5.09	3.30	9.32	8.66
RNA_Polymerase_II_Complex_Pathway	0.13	1.90	0.96	1.71
SMAD_m_Pathway	2.41	3.44	3.30	3.22
SMAD_p_Pathway	2.41	3.44	3.30	3.22
STAT3_Pathway	4.12	3.29	10.90	14.73
TGF_beta_Pathway	0.63	0.71	0.03	0.13
TNF_m_Pathway	0.11	0.35	0.43	0.94
TNF_p_Pathway	0.82	1.52	1.48	0.93
TRAF_m_Pathway	0.10	0.44	0.28	0.35
TRAF_p_Pathway	1.11	1.43	4.46	3.51
Transcription_of_mRNA_Pathway	0.29	1.85	0.65	1.54
Ubiquitin_Proteasome_Pathway	-0.72	2.50	3.01	2.11
Ubiquitination	-0.35	1.23	1.58	1.37
VEGF_Pathway	0.82	1.06	3.19	2.43
WNT	0.33	1.02	-0.20	1.28
Wnt_Pathway	3.76	3.23	8.44	3.84
β-catenin	0.32	0.67	0.30	0.45

Supplementary Table S2. List of investigated geroprotectors and their molecular targets.

Compound_Name	Activation	Inhibition
Nordihydroguaiaretic acid		ALOX12, ALOX15, ALOX5, ATF2, FOS, FOSB, FOSL1, FOSL2, JUN, JUNB, JUND, CES1, ERBB2, Ces1e, FASN, IGF1R, TGFBR1, IFNG, SMAD2
Myricetin		ABCC1, AHR, AKR1B1, CSNK2A1, CSNK2A2, CSNK2B, CDK5, COMT, CYP1A2, IPMK, ITPKA, ITPKB, ITPKC, PIM1, PLK1, POLA1, POLA2, SULT1A1, TOP2A, TOP2B, TTR, PRKCA, PRKCB, PRKCD, PRKCE, PRKCG, PRKCH, PRKCI, PRKCQ, PRKCZ, PRKD1, PRKD2, PRKD3, PRKACA, PRKACB, PRKACG, MYLK, MYLK2, MYLK3, INSR, CSNK1A1, CSNK1A1L, CSNK1D, CSNK1E, CSNK1G1, CSNK1G2, CSNK1G3
HA-1004		AKAP4, CAMK2A, CAMK2B, CAMK2D, CAMK2G, PRKCA, PRKCB, PRKCD, PRKCE, PRKCG, PRKCH, PRKCI, PRKCQ, PRKCZ, PRKD1, PRKD2, PRKD3
7-Cyclopentyl-5-(4-phenoxy)phenyl-7H-pyrrolo[2,3-d]pyrimidin-4-ylamine		SRC, CDK1, EGFR, LCK, PRKCA, PRKCD, PRKCE, PRKCH, PRKCG, PRKCI, PRKCQ, PRKCZ, TEK, KDR, ZAP70
Staurosporine		AKT1, AURKA, ABL1, SRC, CSNK2A1, CSNK2B, CDK1, CDK2, CDK4, CHEK1, PRKCA, PRKCB, PRKCG, EEF1A1, EGFR, SLC29A1, MAPK3, MAPK1, FYN, GSK3B, IKBKB, INSR, LCK, CSF1R, MAP2K1, PDPK1, PIM1, PRKACA, PRKACB, PRKACG, PRKCA, PRKCB, PRKCD, PRKCE, PRKCG, PRKCH, PRKCI, PRKCQ, PRKCZ, PRKD1, PRKD2, PRKD3, PRKD1, PREP, PRKG2, SYK, FLT1, KDR, HTR3A
Fasudil		MYLK, MYLK2, MYLK3, PRKACA, PRKACB, PRKACG, PRKCA, PRKCD, PRKCE, PRKCG
Aspirin		ELANE, ASIC3, ALOX5, PTGS1, PTGS2, IKBKB, SLC22A6, CAPN2
Ursolic acid		PTGS2, SLCO1B1

Compound_Name	Activation	Inhibition
N-acetyl-L-cysteine		AKR1B10, AKR1B1, VKORC1, CSF2RB, MMP2, MMP9, CASP3, CYC1, CHUK, IKBKB, JAK2, MAPK10, NFKB1, NFKB2, REL, RELA, RELB, MAPK11, MAPK12, MAPK13, MAPK14, STAT5A, STAT5B
SB203580		ARAF, ALOX5, BRAF, BMP2, RAF1, SRC, CSNK1D, CCKAR, SLC29A1, GAK, GCGR, MAPK9, LCK, MAPK14, MAPK11, MAPK13, MAPK12, RIPK2, AKT1
Nitrendipine		ADORA1, ADORA2A, ADORA3, CACNA1G, CACNA1I, CACNA2D1, CACNB1, CACNB2, CACNB3, CACNB4, CACNG1, GNRHR, KCNH2, KCNH5, KCNH1, CACNA1C, KCNN4, SLC10A2, TTR, CYP3A4, CACNA1H
Cyclosporin A		SLCO1B1, SLCO1B3, ABCB5, ABCC10, ABCG2, PPP3CA, PPP3CB, PPP3CC, PPP3R1, PPP3R2, PPIA, PPIB, PPIC, PPIE, PPIG, PPIH, CYP3A4, ABCB1, ABCB4, NFATC1, NFATC3, NKTR, PPID, PPIF, PPIL1, YTHDC2, FPR1, SLC10A1, SLCO4C1, Abcb1b, ABCC1, ABCG1, ABCB11, NR3C2, ILF2,ILF3
Wortmannin		MYLK, MYLK2, MYLK3, PLK1, PLK3, AKT1, ATM, PIK3CA, PIK3CB, PIK3CD, PI4KB, SH2D1A
Tyrphostin AG 1478		EGFR, SRC, SLC29A1, FBP1, FBP2, LCK, MAPK14
PP2 AG 1879		ABL1, SRC, SLC29A1, LCK, MAPK14, WNK1, CSNK1D, EGFR
Butein		AKR1A1, ALOX5, SRC, EGFR, IKBKB
LY294002		AKT1, AKT2, AKT3, CSNK2A1, CSNK2A2, CSNK2B, PRKDC, MTOR, PIK3C2B, PDE2A, PDE3A, PDE3B, PIK3CA, PIK3CB, PIK3CD, PIK3CG, PIM1, PLK1
Rosmarinic acid		AKR1A1, FYN
Fisetin		AKR1A1, CDK1, CDK5, CDK6, COMT, CYP1A2, CYP2C9, CYP3A4, GSK3A, GSK3B, HSD17B1, PIM1, PLK1, SULT1A1, TOP2A, TOP2B
Vinpocetine		SCN8A, PDE1A,PDE1B,PDE1C, SCN5A
Indirubin		GSK3A, GSK3B
KN-93		CAMK2A, CAMK2B, CAMK2D, CAMK2G, CAMKK1, CAMKK2, KCNC2, KCNH2, KCNA2, KCNA5, KCNB1, KCND2
1400W		NOS3, NOS2, NOS1
LFM-A13		BTK
Lamotrigine		AQP4, HTR1A, KCNH2, SLC22A1, SCN5A
2-deoxy-D-glucose	PRKAA1,PRKAA2	
2-mercaptoethylamine		QPCT, TGM2
Acarbose		MGAM, SI
AMN082	GRM7	
Amperozide hydrochloride		CHRM1, ADRA1A, ADRA1B, ADRA1D, ADRA2B, DRD2, DRD3, DRD4, DRD5, HRH1, HTR2A, HTR2C
Ascorbic acid		SVCT2
Butylated hydroxytoluene		CAPN1, LDLR
Carbonyl cyanide m-chlorophenyl hydrazone		COX4I1, COX4I2, COX5A, COX5B, COX6A1, COX6A2, COX6B1, COX6B2, COX6C, COX7A1, COX7A2, COX7B, COX7B2, COX7C, COX8A, COX8C, SLC18A1
Carbonylcyanide-p-trifluoromethoxyphenylhydrazone		SLCO1B3
Creatine		GATM
DAPH		EGFR

Compound_Name	Activation	Inhibition
D-chiro-Inositol		GAA, SI, TREH
Deprenyl		CYP2C8, CYP3A4
Dichloroacetic Acid	PPARA	PDK3, BCKDK, PDK1
Didanosine		PNP
Eliprodil		ADRA1A, ADRA1B, ADRA1D, GRIN2B, SIGMAR1
Ethosuximide		ADH1A, ADH1B, ADH1C, CACNA1D, CACNA1G, CACNA1H, CACNA1I, CACNA1S, CACNA1C
Ethylene-diamine-tetra-acetic acid		HIVEP1
Everolimus		FKBP1A, MTOR, SLCO1A2, SLCO1B1, SLCO1B3
GGTI-298		FNTA, PGGT1B
Kanamycin		LYZ
Melatonin	MTNR1A, MTNR1B, RORA, NOS3, G6PD, GPX1, GSR, NOS2, NOS1	CALM1,CALM2,CALM3, CYP1A2, AR
Nicotinamide adenine dinucleotide	QDPR, CLOCK, NPAS2	
Oxaloacetic Acid		DCXR, EGLN1, EGLN2, EGLN3, TST
Sodium butyrate	PTGIR	
Trehalose	TAS1R3	
Valpromide		EPHX1
Vitamin D3		ABCB1
Gallic acid		MMP9, CA1, CA2, CA4, CA9, CA5A, CA5B, DCXR, PNLIP, RAB9A
Juglone		PIN1
Minocycline		SLC25A4, SLC25A5, SLC25A6, CYCS, MMP9, SLC22A6, SLC22A7
1,2,3,4,6-Penta-O-Galloyl-b-D-Glucose		AKR1B10, AKR1B1, AKR1A1, F10, SQLE
Epicatechin		AKR1B10, AKR1B1, BACE1, COMT, PREP, TOP2A, TOP2B, RRM2B
Quercetin-3-O-glucoside		AKR1B1, NQO2
Phosphonoformic acid		CA1, CA2, CA4, CA9, CA5A, CA5B, CA6, CA7, CA8, CA12, CA14, POLA1, POLA2, POLD1, POLD4, POLD2, POLD3, POLG, POLG2
Tamarixetin		ABCC1, AKR1A1
Kenpaullone		CSNK2A1,CSNK2A2,CSNK2B, CDK1, CDK2, CDK5, GSK3A, GSK3B, SIRT2
AG-490		EGFR, SLC29A1, GRIN1, GRIN2A, GRIN2B, GRIN2C, PDGFRA, PDGFRA, CDK2, JAK2, JAK3
Tannic acid		ATE1, CA2, CYP1A1, CYP1A2, MAPT, PRNP
SU 4312		EGFR, ERBB2, IGF1R, PDGFRB, FLT1, KDR
Rapamycin		SLC29A1, ABCG2, FKBP10, FKBP1A, FKBP4, FKBP5, MTOR, MAPKAP1, MLST8, MTOR, RICTOR, SLCO1A2, SLCO1B1, SLCO1B3, ABCB11
Epigallocatechin gallate		BACE1, SLC2A1, MMP7, KDR, COMT, GLUD1, SQLE, FUT7, HDC, IPMK, ITPKA, ITPKB, ITPKC, NAT1, POLA1, POLA2, POLG, PREP, TERT, TOP1, TOP2A, TOP2B, UGT1A4, UGT1A6, SLCO2B1, SLCO1B1, SCN5A, MAPK1, MAPK3, NFKB1, MAPK11, MAPK12, MAPK13, MAPK14
PD-98059		MAPK3, MAPK1, MAP2K5, MAP2K1, MAP2K2, MAP2K3, MAP2K4, MAP2K6, MAP3K1, MAP2K7

Compound_Name	Activation	Inhibition
Tyrphostin AG 1295		KIT, FLT3, PDGFRA, PDGFRB
SP600125		AHR, JAK1, JAK2, JAK3, MAPK8, MAPK9, MAPK10, CDK2, CHEK1, SGK1

Supplementary Table S3. Compound GeroScores for datasets GSE32729 and GSE39540.

Compound_Name	Average score	Average GeroScore for GSE37219	stdev	Average GeroScore for GSE39540	stdev
Nordihydroguaiaretic acid	9.375451485	12.78071429	9.582769	5.970189	9.477559
Myricetin	4.833814017	7.848571429	6.573648	1.819057	4.115186
HA-1004	4.678551213	7.617857143	7.044406	1.739245	3.830018
N-acetyl-L-cysteine	2.511428572	3.852857143	3.885174	1.17	4.945203
7-Cyclopentyl-5-(4-phenoxy)phenyl-7H-pyrrolo[2,3-d]pyrimidin-4-ylamine	2.317702157	3.017857143	5.393759	1.617547	3.042617
Staurosporine	1.841671159	2.114285714	6.47423	1.569057	6.102519
PD-98059	1.549669812	3.565	7.024809	-0.46566	6.581204
Ursolic acid	1.237425876	1.284285714	1.291819	1.190566	1.389004
Fasudil	1.109501348	0.664285714	3.768731	1.554717	2.938064
Aspirin	1.048861186	0.863571429	1.627994	1.234151	1.480605
Epigallocatechin gallate	1.041839623	2.545	4.55105	-0.46132	8.188531
LY294002	1.005896227	1.885	3.464114	0.126792	2.468889
Wortmannin	0.836940701	1.271428571	3.489664	0.402453	2.558764
SB203580	0.677493262	0.388571429	1.153715	0.966415	4.255225
Nitrendipine	0.531799191	0.286428571	2.47638	0.77717	1.53406
Cyclosporin A	0.389946092	0.348571429	0.780432	0.431321	0.854193
Fisetin	0.269083558	0.435714286	0.650369	0.102453	0.434721
PP2 AG 1879	0.253504043	0.132857143	0.353932	0.374151	1.251104
Tyrphostin AG 1478	0.234555256	0.085714286	0.320713	0.383396	1.220872
AG-490	0.210323451	0.478571429	0.953107	-0.05792	1.750546
LFM-A13	0.131785715	0.263571429	0.986194	0	0
KN-93	0.093463612	0.174285714	0.350663	0.012642	0.091175
Vinpocetine	0.068315364	0.052857143	0.135273	0.083774	0.225712
Rosmarinic acid	0.051603774	0	0	0.103208	0.338521
Kenpaullone	0.037001348	0.099285714	0.20428	-0.02528	0.26893
Lamotrigine	0.026428572	0.052857143	0.135273	0	0
Phosphonoformic acid	0.014642857	0.039285714	0.177177	-0.01	0.123464
Indirubin	0.009386793	-0.015	0.056125	0.033774	0.077114
1400W	0.002264151	0	0	0.004528	0.03266
2-deoxy-D-glucose	0	0	0	0	0
2-mercaptoethylamine	0	0	0	0	0
Acarbose	0	0	0	0	0
AMN082	0	0	0	0	0

Compound_Name	Average score	Average GeroScore for GSE37219	stdev	Average GeroScore for GSE39540	stdev
Amperozide hydrochloride	0	0	0	0	0
Ascorbic acid	0	0	0	0	0
Butylated hydroxytoluene	0	0	0	0	0
Carbonyl cyanide m-chlorophenyl hydrazone	0	0	0	0	0
Carbonylcyanide-p-trifluoromethoxyphenylhydrazone	0	0	0	0	0
Creatine	0	0	0	0	0
DAPH	0	0	0	0	0
D-chiro-Inositol	0	0	0	0	0
Deprenyl	0	0	0	0	0
Dichloroacetic Acid	0	0	0	0	0
Didanosine	0	0	0	0	0
Eliprodil	0	0	0	0	0
Ethosuximide	0	0	0	0	0
Ethylene-diamine-tetra-acetic acid	0	0	0	0	0
Everolimus	0	0	0	0	0
GGTI-298	0	0	0	0	0
Kanamycin	0	0	0	0	0
Melatonin	0	0	0	0	0
Nicotinamide adenine dinucleotide	0	0	0	0	0
Oxaloacetic Acid	0	0	0	0	0
Sodium butyrate	0	0	0	0	0
Trehalose	0	0	0	0	0
Valpromide	0	0	0	0	0
Vitamin D3	0	0	0	0	0
Juglone	-0.000283019	0	0	-0.00057	0.053497
1,2,3,4,6-Penta-O-Galloyl-b-D-Glucose	-0.002075472	0	0	-0.00415	0.029938
Epicatechin	-0.002075472	0	0	-0.00415	0.029938
Quercetin-3-O-glucoside	-0.002075472	0	0	-0.00415	0.029938
Tamarixetin	-0.006981132	0	0	-0.01396	0.05757
Tannic acid	-0.041886793	-0.02	0.074833	-0.06377	0.133462
SU 4312	-0.059245283	0	0	-0.11849	3.861833
Butein	-0.085923181	-0.420714286	1.070388	0.248868	0.911943
Gallic acid	-0.129642857	-0.259285714	0.765973	0	0
Minocycline	-0.147506739	-0.291428571	0.763694	-0.00358	0.042341
Rapamycin	-0.20833558	-	0.168531	-0.39453	1.430597

Compound_Name	Average score	Average GeroScore for GSE37219	stdev	Average GeroScore for GSE39540	stdev
		0.022142857			
SP600125	-0.382378706	0.550714286	1.113998	-1.31547	4.282954
Tyrphostin AG 1295	-0.509811321	0	0	-1.01962	2.648521

Supplementary Table S4. Pathway activation analysis results of cellular transcriptional response to NAC, Myricetin and EGCG.

Pathway	MYRICETIN	NAC	EGCG
PAK_Pathway	-2.94	-1.95	1.77
IL_6_Pathway	-2.46	-0.65	0.29
MAPK_Family_Pathway	-2.28	-3.80	0.87
Cellular Senescence	-2.12	-0.60	0.00
TGF_beta_Pathway	-2.10	0.15	1.12
IL_10_Pathway	-1.79	-0.20	0.31
p38_m_Signaling_Pathway	-1.46	-3.72	1.71
ErbB_Family_Pathway	-1.40	0.13	0.15
GSK3_Pathway	-1.38	0.49	1.27
mTOR_Pathway	-1.33	0.09	0.95
VEGF_Pathway	-1.31	-0.36	0.97
Cellular_Anti-Apoptosis_Pathway	-1.17	-0.17	0.66
AKT_Pathway	-1.15	-3.76	1.59
ERK_Signaling_Pathway	-1.10	-2.24	0.41
Chemokine_Pathway	-1.07	-0.58	0.37
TRAF_p_Pathway	-0.94	0.65	0.27
SMAD_m_Pathway	-0.92	1.24	-0.25
SMAD_p_Pathway	-0.92	1.24	-0.25
Growth_Hormone_Pathway	-0.92	-0.20	0.00
Inflammation	-0.91	0.49	0.00
NFkB	-0.91	0.00	0.27
Cytokine_Network_Pathway	-0.91	-0.22	0.00
FLT3_Signaling_Pathway	-0.89	-0.30	0.45
Erythropoeitin_Pathway	-0.83	-1.06	0.23
p53_Signaling_m_Pathway	-0.79	1.57	-0.44
STAT3_Pathway	-0.73	-1.46	0.78
Integrin_SIgnaling_Pathway	-0.72	-2.61	-0.06
EGFR1_Pathway	-0.64	1.55	0.00
JNK_Pathway	-0.58	-0.59	1.52
HGF_Pathway	-0.57	-0.90	-0.27

Pathway	MYRICETIN	NAC	EGCG
GPCR_Pathway	-0.49	-1.18	1.29
TRAF_m_Pathway	-0.48	0.00	0.00
Heat shock response	-0.43	-0.57	0.00
RANK_Signaling_in_Osteoclast_Pathway	-0.42	0.49	0.00
WNT	-0.39	0.10	-0.42
PI3K-AKT	-0.37	0.00	0.00
Estrogen_Pathway	-0.35	-1.17	0.52
IP3_Pathway	-0.34	-0.65	1.19
Mismatch_Repair_Pathway	-0.33	-0.34	-0.46
ER stress response	-0.32	-0.24	0.00
CD40_Pathway	-0.31	-0.21	0.00
NGF_p_Pathway	-0.29	-0.54	0.39
NRF2 Oxidative Stress Response	-0.28	-0.49	0.35
Fas_m_Signaling_Pathway	-0.24	-0.57	-0.37
IGF-1	-0.23	0.38	0.00
Circadian_Pathway	-0.20	-0.53	0.00
Nucleotide Excision Repair	-0.17	0.00	0.19
Hedgehog_Pathway	-0.16	-0.25	0.31
MAPK_Signaling_Pathway	-0.16	-2.57	1.77
Polycomb-Trithorax	-0.10	-1.33	0.00
CREB_Pathway	-0.05	-1.41	1.67
Androgen_receptor_Pathway	-0.01	-0.34	1.28
Notch	0.00	0.00	1.20
Hypoxia-induced_EMT_in_cancer_and_fibrosis_3	0.00	0.00	0.81
DNA Methyltransferases	0.00	0.00	0.00
Interactions Report	0.00	0.00	0.00
β-catenin	0.00	0.00	0.00
Double-Strand Break Repair	0.00	-0.04	0.00
Histone Deacetylases	0.00	-0.19	0.22
Mismatch Repair	0.00	-0.34	0.21
Notch_Pathway	0.00	-0.35	1.03
NGF_m_Pathway	0.00	0.37	0.27
Fas_p_Signaling_Pathway	0.02	0.00	0.50
Hippo	0.10	0.00	0.33
TNF_m_Pathway	0.10	-0.57	0.29
Chromatin_Pathway	0.11	-0.39	0.00
JAK_mStat_Pathway	0.15	0.00	0.00
Hedgehog	0.21	-0.25	0.20
Autophagy	0.22	0.47	0.00
Caspase_Cascade	0.24	0.97	-0.37

Pathway	MYRICETIN	NAC	EGCG
Osmotic Stress	0.32	-0.18	0.00
Ubiquitination	0.36	0.26	0.24
Glucocorticoid_Receptor_Pathway	0.37	-0.50	0.90
DNA damage response	0.46	0.00	0.00
Mitochondrial_Apopotosis_m_Pathway	0.48	0.78	-1.16
eIF4e-p70 S6	0.50	0.00	0.00
mTOR	0.50	0.00	0.00
ATM_Pathway	0.58	-0.31	0.61
PTEN_Pathway	0.63	0.57	0.00
IL_2_Pathway	0.64	1.01	-0.07
TNF_p_Pathway	0.67	0.49	0.50
Circadian Rhythms	0.68	-0.53	0.00
RAS_Pathway	0.75	-2.36	-0.64
Oxidative Stress Response	0.76	-0.52	0.00
Base Excision Repair	0.85	0.00	0.00
PPAR_Pathway	1.05	-0.97	0.34
Transcription_of_mRNA_Pathway	1.06	-0.20	0.26
RNA_Polymerase_II_Complex_Pathway	1.06	-0.20	0.00
Wnt_Pathway	1.12	-1.69	2.22
Antioxidants	1.28	-0.15	0.00
Ubiquitin_Proteasome_Pathway	1.39	-1.16	0.42
IGF1R_Signaling_Pathway	1.40	-0.23	0.43
HIF1Alpha_Pathway	1.50	0.26	0.35
Hypoxia	1.72	-1.02	0.00
cAMP_Pathway	1.75	-1.17	2.70
DNA_Repair_Mechanisms_Pathway	1.79	0.14	-0.29
ILK_Pathway	2.38	0.94	2.35

Supplementary Table S5. DNN-based side effects probabilities of investigated compounds.

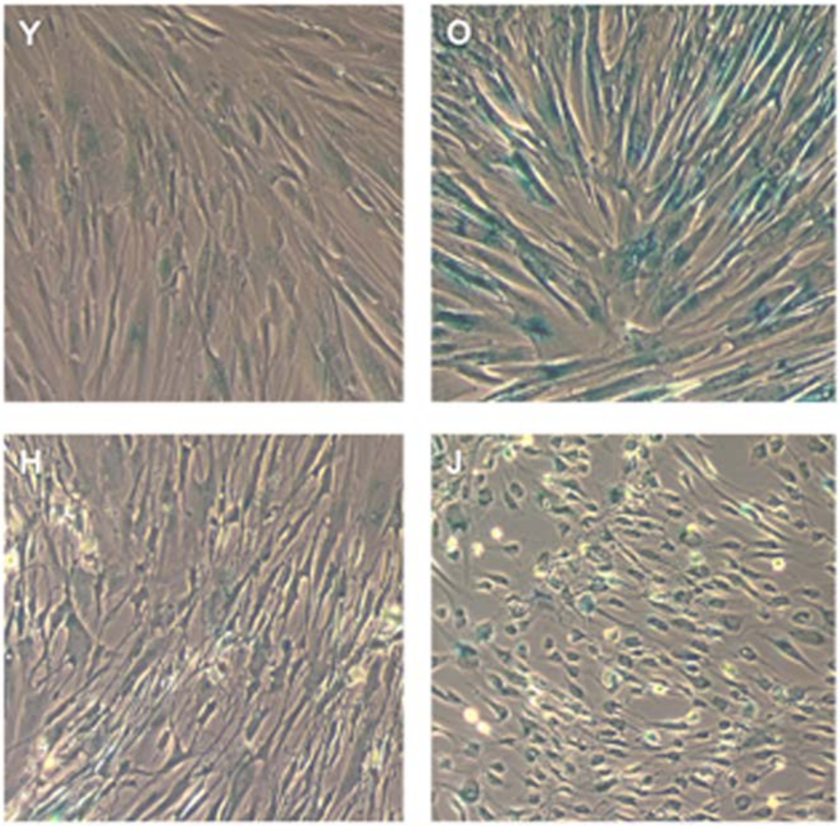

Supplementary Figure S1. High magnification images of cell morphology of young, old, Fasudil- and EGCG-treated senescent fibroblasts. Group letter codes are listed in Table 1.
